# Early identification and prevention of cardiometabolic risk in Gulf Cooperation Council countries: a scoping review relevant to primary care and family medicine

**DOI:** 10.1016/j.pmedr.2026.103662

**Published:** 2026-10-12

**Authors:** Aseel Awad Alsaidan, Bashayer Farhan Alruwaili, Muhannad Almubarak, Abdulaziz Ibrahim Alzarea, Hassan H. Alhassan, Ali Farhan Alanazi, Rami Hamdan Alharbi, Ali M. Alzweihary

**Affiliations:** a Department of Family and Community Medicine, College of Medicine, Jouf University, Sakaka, Aljouf, Saudi Arabia; b Department of Internal Medicine, College of Medicine, Jouf University, Sakaka, Aljouf, Saudi Arabia; c Department of Clinical Pharmacy, College of Pharmacy, Jouf University, Sakaka, Aljouf, Saudi Arabia; d Department of Clinical Laboratory Sciences, College of Applied Medical Sciences, Jouf University, Sakaka, Aljouf, Saudi Arabia; e Department of Cardiology, Aljouf Cardiac Center, Aljouf Health cluster, Sakaka, Aljouf, Saudi Arabia; f Department of Family and Community Medicine, Prince Sultan Military Medical City, Riyadh, Saudi Arabia; g Department of Medicine, College of Medicine, Qassim university, Qassim, Saudi Arabia

**Keywords:** Diabetes, Primary care, Obesity, Cardiometabolic, Screening, Prevention, Gulf Cooperation Council countries

## Abstract

**Objectives:**

This scoping review was aimed at mapping and identifying the current evidence on the early identification and prevention of cardiometabolic risks in Gulf Cooperation Council countries, in the context of primary care and family medicine, and to identify where evidence is concentrated or absent.

**Methods:**

Original studies in English were searched in PubMed/MEDLINE, Scopus, and Web of Science, between January 2020 and February 2026. The eligible studies were conducted in Gulf Cooperation Council countries and focused on cardiometabolic risk, early identification, screening, prevention, risk reduction, lifestyle interventions, health education, or preventive strategies. Data were charted using a structured form and synthesised descriptively.

**Results:**

The evidence indicated that the high and clustered burden of dysglycemia, prediabetes, diabetes, metabolic syndrome, hypertension, dyslipidemia, and central obesity existed among adults, children, adolescents, university students, community populations, primary care settings, and biobank-based studies. Studies demonstrated the value of risk scores, anthropometric measurements, biomarkers, and community screening strategies. Prevention and early-intervention studies were less frequently represented and heterogeneous across settings.

**Conclusion:**

Current Gulf Cooperation Council evidence supports the importance of early cardiometabolic risk identification and prevention but also highlights the need for integrated prevention pathways within primary care and family medicine.

## Highlights

• Cardiometabolic risk studies in Gulf Cooperation Council countries are mapped.
• Obesity, diabetes, hypertension, dyslipidemia, and lifestyle are key risks.
• Primary care supports early screening, risk assessment, and prevention.
• Gaps remain in regional evidence, implementation, and long-term prevention.
• Stronger primary care may improve detection and reduce disease burden.

## 1 Introduction

Cardiovascular diseases are the leading cause of mortality in the world, and noncommunicable diseases in general present the majority of premature deaths in adults (WHO, 2025a). Clinically, the cardiometabolic risk concept is particularly advantageous since it describes the aggregation and interplay of obesity, dysglycemia, high blood pressure, dyslipidemia, and other metabolic abnormalities, which usually precede and follow the onset of overt cardiovascular disease (Peterseim et al., 2024).

Obesity is closely associated with hypertension, type 2 diabetes, dyslipidemia, and downstream cardiovascular disease, whereas metabolic syndrome remains a convenient clinical construct that is able to identify people with an increased risk profile (Yamada et al., 2023; Zheng et al., 2024). Contemporary studies also indicate that the prevention of nutrient isolation or individual risk factors should not be considered; rather, it should be based on more general patterns of diet, physical activity, weight control, and multi-component lifestyle approaches (Rutters et al., 2024).

These facts put primary care and family medicine at the core of cardiometabolic prevention (Sterling et al., 2024; Bam et al., 2024). The initial health care provider for asymptomatic or minimally symptomatic adults with excess weight, elevated blood pressure, abnormal glucose, unhealthy lipid profiles, or a combination of risk factors is often a family physician (Sterling et al., 2024). Recent preventive recommendations emphasize the role of risk evaluation, counseling, follow-up over the period, and early management in routine practice, rather than late intervention when complications arise (Jones et al., 2025). Recent primary care work also focused on the need for better quality indicators, realistic approaches to obesity pathways, and clinically applicable methods for identifying and treating metabolic syndrome (Bam et al., 2024).

The Gulf Cooperation Council countries are the setting that is especially significant for such a review (Alqadasi et al., 2024; Fadhil et al., 2022). The area has experienced a fast demographic, nutritional, and lifestyle change, with high rates of obesity, diabetes, and other noncommunicable diseases (Alqadasi et al., 2024; Fadhil et al., 2022). Recent studies of the region indicate significant and unremitted morbidity of type 2 diabetes mellitus and obesity, and larger-scale reviews of Gulf Cooperation Council health systems and disease burden indicate that prevention-based responses are an immediate concern (Alqadasi et al., 2024; Fadhil et al., 2022). Gulf Cooperation Council-specific studies also provide evidence that upstream prevention opportunities are not limited to adult clinic care alone but also include school-based and population-level approaches, all of which are highly applicable to the long-term practice of family medicine (Alzahrani et al., 2025). Simultaneously, the burden is not uniformly distributed, and the region's different health systems, population patterns, and implementation capabilities might influence the provision of early identification and prevention (Alqadasi et al., 2024; AlMarzooqi et al., 2023).

Previous regional syntheses have examined the prevalence of metabolic syndrome across Gulf Cooperation Council countries, national health-system responses to noncommunicable disease management, school-based weight-related interventions, and obesity-related cardiovascular risk in the Arab Gulf (Fadhil et al., 2022; Shin and Jee, 2020; Banany et al., 2024). Although these reviews provide important evidence on disease burden, health-system responses, or specific risk factors and populations, they do not collectively map early identification and prevention across multiple cardiometabolic risk domains, life-course groups, and community and healthcare settings from a primary care and family medicine perspective. Therefore, this scoping review aims to map the current evidence on early identification and prevention of cardiometabolic risk in Gulf Cooperation Council countries, with relevance to primary care and family medicine, and to describe relative evidence concentrations and absences to guide future research and practice. Accordingly, the review was guided by the following Population–Concept–Context -framed question (Peters et al., 2020): “What evidence exists regarding the early identification and prevention of cardiometabolic risk among individuals and population groups in Gulf Cooperation Council countries across public health, primary care, and family medicine-relevant settings?”

## 2 Methods

### 2.1 Review framework and registration

The present scoping review was conducted in accordance with the Preferred Reporting Items for Systematic Reviews and Meta-Analyses extension for Scoping Reviews and registered with Open-Science Framework (Registration link: https://doi.org/10.17605/OSF.IO/N9FGC).

### 2.2 Eligibility criteria

Included in studies were those carried out in a Gulf Cooperation Council country or countries; that discussed cardiometabolic risk factors, early cardiometabolic conditions, early identification, screening, prevention, risk reduction, lifestyle intervention, health education, or preventive methods. Original research with human subjects or population-level data was eligible. Studies in English, that are observational, interventional, and mixed methods were included. The English-language restriction was applied because translation resources were unavailable. Only peer-reviewed journal articles were included; grey literature, including reports, policy documents, theses, and preprints, was excluded. The studies were excluded in case they were done on management of established disease without any relation to early detection or prevention or were on a condition that lacked a clear cardiometabolic component, or were review articles, editorials, commentaries, conference abstracts, protocols, letters, or case reports and outside the included region.

### 2.3 Information sources and search strategy

A thorough search was done in PubMed/MEDLINE, Scopus, and Web of Science. The publication-date eligibility period was January 1, 2020, through February 28, 2026. Although the databases were searched till March 31, 2026, articles published after February 28, 2026, were not eligible for inclusion. This timeframe was selected to capture the most recent and practically relevant evidence reflecting current preventive priorities and primary care practice. No additional information sources or supplementary search methods, such as citation chasing or manual searching, were used. The Boolean operators such as AND/OR/NOT were used to combine keywords, and in the case of controlled vocabulary, the operators were used. The search terms were cardiometabolic risk, obesity, hypertension, dyslipidemia, metabolic syndrome, prediabetes, type 2 diabetes, and insulin resistance; screening, early detection, prevention, risk assessment, health promotion, and lifestyle modification; and regional terms, such as Gulf Cooperation Council, Saudi Arabia, United Arab Emirates, Kuwait, and so on. Each database was searched based on its search interface and indexing system. The complete database-specific search strategies are provided in Supplementary Table S1.

### 2.4 Data charting and synthesis

A structured form was used to chart relevant data from the included studies. The extracted data were the author, year of publication, country, and study design, population characteristics, setting, cardiometabolic risk focus, type of early identification or prevention measure, and key findings. The charting form was piloted on a subset of included studies and refined before full data charting and adhered to Population–Concept–Context. Similar measures were grouped into broader themes, and studies could contribute to more than one theme. Following duplicate removal, two reviewers independently conducted title-and-abstract screening, full-text screening, and data charting; disagreements at any stage were resolved through discussion and, when necessary, consultation with a third reviewer. The synthesis of the findings was conducted in a descriptive, narrative way, considering the distribution of evidence across countries, populations, settings, and prevention themes, and how these relate to primary care and family medicine. The narrative synthesis was informed by Popay et al. (Popay et al., 2006). Reasons for full-text exclusion were recorded and are summarised in Fig. 1. A priori mapping domains comprised population and life stage, setting, cardiometabolic risk focus and outcomes, identification and screening, and prevention and early intervention. The distribution across these domains was interpreted against contemporary prevention guidance, with absences treated as relative to the literature located rather than definitive wide gaps in the region (Sterling et al., 2024; Ndumele et al., 2023; Lloyd-Jones et al., 2022). No formal critical appraisal or risk-of-bias assessment was undertaken because the review aimed to map the breadth and nature of the evidence rather than evaluate intervention effectiveness.

**Fig. 1:**
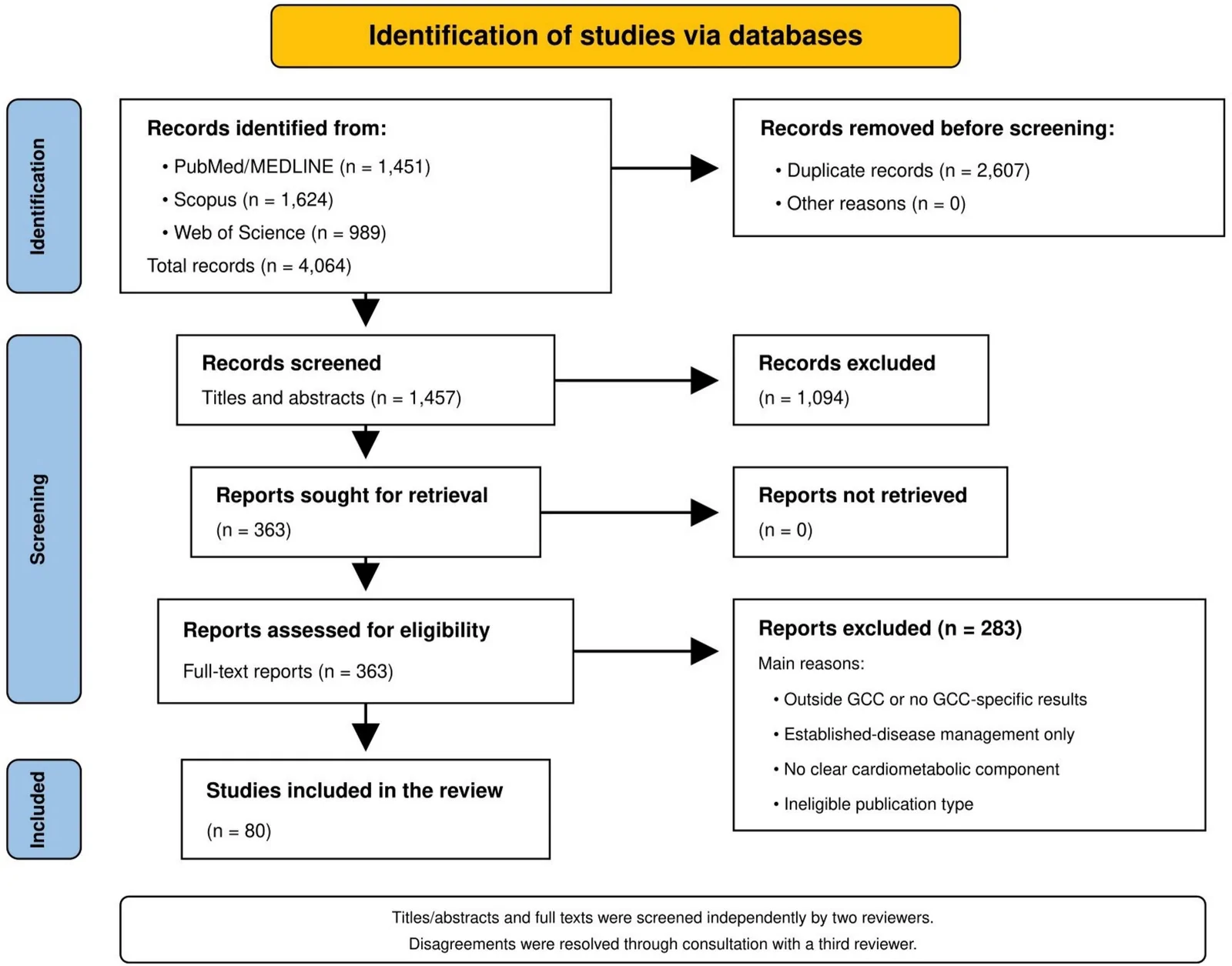
Study selection for a scoping review of early identification and prevention of cardiometabolic risk among children, adolescents, adults, and healthcare professionals in Gulf Cooperation Council countries, with studies published January 2020 to February 2026.

## 3 Results

### 3.1 Overview of included studies

The database search identified 4064 records. After 2607 duplicates were removed, 1457 records were screened, of which 1094 were excluded. All 363 reports sought were retrieved and assessed for eligibility; 283 were excluded mainly because they were outside or lacked Gulf Cooperation Council-specific results, addressed established-disease management only, lacked a clear cardiometabolic component, or were ineligible publication types, leaving 80 included studies (Fig. 1). Studies were conducted in Saudi Arabia (*n* = 31), the United Arab Emirates (*n* = 13), Qatar (*n* = 13), Kuwait (*n* = 12), Oman (*n* = 8), and Bahrain (*n* = 3). The populations of the studies were heterogeneous and comprised of children, adolescents, university students, community adults, biobank participants, primary care attendants, healthcare workers, and primary care physicians. Table 1 summarizes the characteristics and approaches represented across the included studies, while Supplementary Table S2 provides study-level characteristics and key findings on early identification and prevention of cardiometabolic risk in Gulf Cooperation Council countries.

**Table 1:** Distribution and characteristics of studies on early identification and prevention of cardiometabolic risk among children, adolescents, adults, and healthcare professionals in Gulf Cooperation Council countries, published January 2020 to February 2026.

Characteristic	Overview of included studies
Country distribution	Saudi Arabia, 31; United Arab Emirates, 13; Qatar, 13; Kuwait, 12; Oman, 8; Bahrain, 3. Total: 80 studies.
Study designs	Predominantly cross-sectional surveys and analyses of health records, surveillance data, or biobanks. Other designs included longitudinal cohorts, case-control studies, risk-score development and validation, mathematical modelling, and a pilot intervention.
Population groups	Community adults; primary care patients; adults with obesity or elevated diabetes risk; children and adolescents; university students; and healthcare professionals.
Settings	Community and national surveys, biobanks, schools and universities, primary care centres, hospitals, lifestyle medicine clinics, and community screening programmes.
Cardiometabolic risk domains	Excess weight and central adiposity; dysglycaemia and diabetes risk; metabolic syndrome; blood pressure; lipids; and related dietary, physical activity, and lifestyle factors.
Early identification and prevention approaches	Risk scores, anthropometric measures, biomarkers, opportunistic and community screening, awareness and health education, and early lifestyle intervention.

### 3.2 Patterns of cardiometabolic risk relevant to primary care and family medicine

In Saudi Arabian, Qatari, Kuwaiti, United Arab Emirates, Omani, and Bahraini studies of adults, dysglycemia, prediabetes, diabetes, obesity, metabolic syndrome, central obesity, hypertension, and dyslipidemia were found to be prevalent with age and usually accompanied by other risks and not found as isolated conditions (S1- S23). Qatari and United Arab Emirates studies also highlighted metabolic perturbations associated with obesity, unfavourable lipid phenotypes, and a wider cardiometabolic disaggregation of both community and biobank groups (S20, S24 – S30). Excess body weight, central adiposity, dysglycemia, hypertension, and dyslipidemia were consistently found to accumulate among young adults in the United Arab Emirates Healthy Future Study and associated analyses, supporting the importance of risk awareness and detection at early life and early adulthood in health care environments (S6, S12, S30, S31).

The burden was also reflected in the younger age groups, where there were a number of studies that reported significant levels of overweight, obesity, central obesity, impaired glucose regulation and obesity-related metabolic risk among children, adolescents, and students (S32 – S45). The risk of obesity was also associated with abnormal glycemia, adverse cholesterol patterns, blood-pressure increase, reduced levels of high-density lipoprotein cholesterol, and general metabolic abnormalities in both young and adult groups (S6, S10, S17, S25, S28, S33, S41, S46). Combined, these findings suggest that cardiometabolic risk in Gulf Cooperation Council countries is not confined to definite disease groups but is already manifested throughout the life course in community, school, university, and adult-care environments, thus being extremely important to the practice of primary care and family medicine (S1, S2, S4, S9, S16, S19)**.**

### 3.3 Early identification, screening, prevention, and intervention approaches

A number of research studies constructed or confirmed non-invasive risk scores and practical screening instruments, such as a Saudi dysglycemia risk score, the Arabic version of the American Diabetes Association Prediabetes Risk Test, Qatar-specific diabetes and prediabetes risk scores, and a revised Omani diabetes screening score (S1, S26, S29, S47–S51). These studies supported the utility of age, anthropometry, blood pressure, family history, and other clinical factors to identify high-risk individuals in a viable manner prior to the establishment of overt disease (S1, S26, S29, S47, S49, S50, S52).

Other studies focused on anthropometric and biomarker-based screening methods, demonstrating the clinical usefulness of waist circumference, waist-to-height ratio, the visceral adiposity index, ethnicity-specific anthropometric cut-points, salivary biomarkers, triglyceride-based risk markers, handgrip strength-related associations, and atherogenic measures to identify individuals at increased cardiometabolic risk (S17, S24, S25, S45, S46, S53 – S56). The opportunity to detect previously unknown cases of diabetes or prediabetes outside of traditional diagnostic processes was also supported by community and opportunistic screening programs, such as text-message-based screening-based screening in Kuwait and community-based diabetes and prediabetes screening in Saudi Arabia (S52, S57). Simultaneously, a Omani and Qatar national or modeled screening setting indicated that earlier detection is possible at the population level with the assistance of a structured screening instrument and specific risk forecasting models (S23, S48-S50). Overall, the evidence reviewed indicates that the Gulf Cooperation Council already has several implementable methods for earlier identification of cardiometabolic risks, but these are more often assessed as tools or models rather than as part of a comprehensive prevention pathway (S1, S26, S47, S57).

Prevention methods used in the studies included those in the fields of health education, assessment of awareness, risk communication, lifestyle counseling, prevention-oriented recruitment, culturally acceptable and early-stage lifestyle intervention (S58 – S68). The awareness of risk factors of coronary artery diseases, knowledge of prediabetes, and diabetes prevention in adults at higher risk have been investigated in Saudi Arabia and the United Arab Emirates, with gaps in knowledge being demonstrated, but early intervention in terms of weight- and waist-related outcomes is possible with the help of education (S58, S59, S66).

Other studies also focused on perceptions and intervention-related behavior opportunities, including obesity perceptions in Bahraini adults and lifestyle medicine in obese adults in primary care-based contexts (S65, S67).

### 3.4 Population groups and contextual factors influencing cardiometabolic risk

A large proportion of the literature has been on children, adolescents, and young adults, such as school children, university students, female undergraduates, and medical students, indicating that overweight, obesity, central obesity, impaired fasting glucose, and other metabolic abnormalities tend to manifest themselves earlier in life (S32, S33, S39, S42, S44, S45, S69 – S74). Cardiometabolic risk was also found in studies in these groups in association with behavioral and environmental factors, including breakfast skipping, irregular meals, sugar-sweetened beverages, screen time, physical inactivity, sleep patterns, and parental obesity or family history (S32, S33, S64, S75)**.**

Contextual factors in adults, such as sex, age, obesity, family history, education, occupation, marital status, nationality, and ethnic subgroup differences, were correlated with different cardiometabolic patterns in settings (S5, S21, S56, S76). A few Saudi, United Arab Emirates, Qatar, and Oman studies also pointed to the existence of the uneven load of the burden among the subgroups, as there were variations in the burden by region, nationality, body-composition profile, or family history of cardiovascular diseases (S11, S19, S28, S30). Other research on obesity awareness, community knowledge, and the perceived body weight further revealed that social perception and health awareness can be used to influence the context of prevention (S58, S60, S63, S65). This evidence suggests that cardiometabolic prevention in Gulf Cooperation Council countries should be adjusted to life stage, sex, behavior, family history, and social context of the country, as opposed to providing a consistent model that should fit all populations (S30, S32–S34, S77).

### 3.5 Evidence relevant to primary care and family medicine practice

The most directly applicable evidence came from primary healthcare centers, electronic medical record-based primary care datasets, physician surveys, primary care validation studies, and lifestyle medicine clinics (S2, S15, S49, S67, S78 – S80). These studies demonstrated the opportunities of primary care and family medicine to act as effective platforms of opportunistic screening, prediabetes and obesity identification, risk stratification, counseling, and early follow-up, in the case of simple non-invasive tool support and locally applicable risk scores (S2, S26, S29, S49). They also emphasized the fact that the evidence of prevention in the context of frontline care does not solely focus on the patient, as the survey of primary care doctors in Saudi Arabia demonstrated gaps in knowledge, practice variability, and possible improvement of screening and management of prediabetes and associated cardiometabolic risk (S77, S79).

Among the studies located, few evaluated integrated systems linking screening, counseling, referral, and long-term follow-up within family medicine (S2, S29, S49, S57, S67).

## 4 Discussion

The analyzed literature rather supports the larger perspective that early risk conditions, behavioral determinants, and contextual factors already have a significant role to play before overt disease manifestation into clinical recognition (WHO, 2025b). This especially holds true in a place where obesity, dysglycemia, hypertension, and other risk profiles tend to be formed in settings that are influenced by high rates of urbanization, sedentary living, change in diet, and other structural factors (Aljulifi, 2021). In this regard, the evidence highlights the need to move beyond downstream disease management to earlier risk identification and preventive action (Visseren et al., 2021).

Existing works of evidence were more robust in establishing the presence and distribution of cardiometabolic risk compared to showing the systematic implementation, maintenance, or integration of preventive efforts into routine care (Uchmanowicz et al., 2025). These findings thus favor the perspective that cardiometabolic prevention in Gulf Cooperation Council countries is not merely a question of defining high-risk groups, but also of enhancing the systems, environments, and care designs within which early detection can result in significant intervention (Uchmanowicz et al., 2025).

Collectively, these results indicate that the available literature offers an effective starting point for prevention-oriented planning, yet they also demonstrate that there is a necessity to enhance the level of coherence in the conceptualization and approach cardiometabolic risk across the region (Ndumele et al., 2023). This interpretation is particularly relevant in the context of a primary care and family medicine viewpoint since these two fields are ideally placed to bridge the gap between risk assessment, continuity of care, lifestyle counseling, and early intervention throughout the life course.

According to broader primary care literature, the Gulf Cooperation Council evidence is mostly aligned in recognizing primary care and family medicine as the most suitable environments for early cardiometabolic risk work (Habte-Asres et al., 2024). The reason is that prevention models used internationally already involve risk assessment, lifestyle counseling, and longitudinal follow-up as a component of first-contact care, and Saudi primary care recommendations have also structured cardiometabolic prevention as a component of patient-centered risk assessment, lifestyle change, chronic care planning, and follow-up instead of disease treatment alone.

A large part of the local literature has done better at describing the burden and clustering of cardiometabolic risk than at illustrating fully integrated, daily primary care prevention pathways (Amir et al., 2023). This is in line with the wider Gulf Cooperation Council evidence of the high prevalence of metabolic syndrome and intersecting risk conditions, and Saudi data also suggest that screening attendance to blood pressure, cholesterol, and cardiovascular risk can remain low, and that compliance with obesity and hypercholesterolemia prevention advice among some primary healthcare physicians remains low (Shin and Jee, 2020; Amir et al., 2023; Gosadi, 2021). By contrast, cardiometabolic prevention is more frequently talked about in the wider primary care literature as a form of organized continuum of risk stratification, counseling, and follow-up (Amir et al., 2023).

Multidisciplinary, behavior-oriented care is becoming the new standard for cardiometabolic prevention internationally, with referral or counseling pathways centered in primary care (Lindblom et al., 2024; Tu et al., 2024). The same seems to be the direction that the Gulf Cooperation Council literature takes, although there is still no integration (Lindblom et al., 2024; Jairoun et al., 2023). For example, one of the United Arab Emirates studies demonstrated that screening for diabetes and cardiovascular risk by community pharmacies was feasible, that a substantial number of at-risk adults were identified, and that participants were very satisfied; however, the authors also noted that collaboration with primary care physicians needed to be developed.

The wider prevention literature is now beginning to embrace the shift towards an integrated cardiometabolic risk assessment as an alternative to isolated disease-based screening, and the implication of the Gulf Cooperation Council evidence seems to be homogenous (Lloyd-Jones et al., 2022). Instead of screening for obesity, dysglycemia, hypertension, and dyslipidemia as distinct issues, it would be more beneficial to consider these as overlapping risk areas that frequently co-occur and which should be identified at an earlier stage before the actual disease manifestation occurs. This finding aligns with Saudi cardiometabolic risk guidelines, which position primary care assessment based on a combination of risk assessment and longitudinal follow-up and with local policy reports highlighting cardiovascular and diabetes-risk screening as an element of preventive care as opposed to a late-stage reaction to established morbidity (Alhabeeb et al., 2025).

The second implication is that the benefits of screening are limited if it is not coupled with established follow-up and intervention routes (Force USPST, 2021). This issue is supported by Saudi statistics of low adoption of regular health check-ups, low modeled adoption of cardiovascular and diabetes risk screening, and records of poor compliance with obesity-prevention measures among a section of primary healthcare practitioners (Gosadi, 2021; Aljaaly and Khalifa, 2023).

The available literature also indicates that early intervention must not be limited to physician-led clinical interactions only (Motlohi et al., 2023). Among the more useful lessons of recent Gulf Cooperation Council work is that opportunistic and community-facing models could expand the entry points for prevention, especially where uptake of routine screening is low.

Extensive evidence is in favor of lifestyle intervention in individuals with high cardiometabolic risk and demonstrates that structured interventions can delay or slow the onset of type 2 diabetes mellitus and improve several cardiometabolic measures (Rosenfeld et al., 2025). Within the Saudi context, though, the research in prediabetes management indicates that preventive pharmacotherapy like metformin is underutilized even among high-risk individuals who are eligible and that primary care physicians may have gaps in their knowledge or practice in prediabetes. In that regard, the implications of this review are more in favor of a more proactive model where screening is repeated, risk stratified earlier, and preventive action taken before it is too late and the established chronic disease has developed (Rosenfeld et al., 2025; American Diabetes Association Professional Practice C, 2024).

Despite the common high cardiometabolic risk burden and numerous overall health-system similarities, the six countries have diverse population structures, service organization, financing patterns, and the extent to which prevention policies have been enacted (Fadhil et al., 2022). It implies that cardiometabolic prevention in the Gulf Cooperation Council is defined not only by disease burden but also by demographic diversity, workforce, and disparities in how preventive care is applied in practice across countries (Fadhil et al., 2022).

A systematic review on the subject of women in Gulf Cooperation Council countries reported that inactivity is not entirely the responsibility of individual motivation but also other factors, including lack of social support and perceived incompetence. The trends indicate that the prevention approaches in the area might have to be more culturally and socially specific, especially in cases when lifestyle change is likely to be a key element of early cardiometabolic risk mitigation (Fadhil et al., 2022; Alzahrani et al., 2025).

Reviews of this region's health systems indicate that preventive measures have been introduced to varying degrees, including legislative, educational, and health-promotion approaches, but that many of these measures depend on sectors outside health care. This is especially relevant for cardiometabolic risk because the major prevention drivers extend beyond the clinic into food environments, education, transport, and opportunities for active living (Banany et al., 2024; Albujulaya et al., 2024).

Evidence was concentrated on risk burden and screening, whereas fewer studies addressed integrated prevention, linkage to care, referral, and sustained follow-up. These findings represent relative absences within the literature located by this review, rather than definitive wide gaps in the region.

Future studies in this area would be enhanced by better conceptualization of cardiometabolic risk across studies, greater consistency in the outcomes reported, and a more explicit focus on how various domains of risk are measured.

Another significant direction for the future is the production of evidence more relevant to primary care and family medicine. Future research needs to better address how prevention can be incorporated into first-contact care, how at-risk patients can be identified during routine interactions, and how preventive measures can be adapted to various age groups, care environments, and risk profiles.

Finally, future research should pay greater attention to regional diversity. The field would be strengthened, therefore, by more prospective, interventional, and implementation-oriented research that can move the field beyond risk identification to evidence that better informs actual prevention planning.

Some limitations need to be noted while interpreting the present review's findings. To start with, since this was a scoping review, the purpose was not to quantitatively estimate intervention effectiveness or produce pooled estimates of the evidence, but to map the availability and nature of the available evidence. Second, the articles included were diverse concerning populations, outcomes, settings, and preventive focus, which restricted direct comparisons across studies. Third, only the studies that were written in English were included in the review, which might have left some regional literature behind. In addition, the search was also limited to three databases and the period from January 2020 to February 2026 and did not include grey literature or policy documents. The identified absences should therefore be interpreted within these limits. These restrictions may also have introduced database-selection and search-related bias. Moreover, the uneven representation of countries limits the extent to which the findings represent the region as a whole. Although two reviewers independently conducted the screening, reviewer judgement during study selection, data extraction, and thematic classification may have introduced selection or extraction bias. Furthermore, the absence of formal critical appraisal also means that the findings should not be interpreted as an assessment of the methodological quality or certainty of the evidence included. Lastly, the predominantly descriptive and observational evidence did not permit comparison of intervention effectiveness.

## 5 Conclusion

Collectively, this scoping review demonstrates a necessity to shift the focus from describing cardiometabolic burden to more inclusive and implementable prevention pathways in the Gulf Cooperation Council countries. The mapped evidence was predominantly descriptive and observational, with unequal representation across the countries and limited evaluation of integrated screening and preventive interventions. The results support the applicability of primary care and family medicine as the primary screening, risk evaluation, counseling, and early intervention platforms, as well as the impact of regional and contextual variables on the ability to provide prevention effectively. At the policy level, standardised risk-assessment pathways, culturally appropriate interventions, stronger referral systems, and integration of prevention into routine primary care should be prioritised. Finally, there is a need for more coherent, implementation-oriented, and context-responsive research. Future studies should particularly address underrepresented countries and populations and evaluate scalable interventions using comparable outcomes. This could strengthen the evidence base and support preventive strategies that are practical, scalable, and meaningful for Gulf Cooperation Council health systems.

## Declaration of generative AI and AI-assisted technologies in the manuscript preparation process

During the preparation of this work, the author(s) used ChatGPT and Grammarly for assistance with language enhancement, grammar checking, and improvement of manuscript readability. The author(s) reviewed and edited the output as needed and take full responsibility for the content of the published article.

## CRediT authorship contribution statement

**Aseel Awad Alsaidan:** Writing – original draft, Validation, Resources, Methodology, Funding acquisition, Conceptualization. **Bashayer Farhan Alruwaili:** Writing – original draft, Visualization, Project administration, Methodology, Investigation, Funding acquisition, Conceptualization. **Muhannad Almubarak:** Writing – original draft, Visualization, Validation, Methodology, Investigation, Conceptualization. **Abdulaziz Ibrahim Alzarea:** Writing – original draft, Software, Resources, Methodology, Investigation, Data curation. **Hassan H. Alhassan:** Writing – review & editing, Visualization, Resources, Methodology, Investigation, Data curation. **Ali Farhan Alanazi:** Writing – review & editing, Visualization, Project administration, Methodology, Conceptualization. **Rami Hamdan Alharbi:** Writing – review & editing, Visualization, Methodology, Investigation, Conceptualization. **Ali M. Alzweihary:** Writing – review & editing, Visualization, Resources, Methodology, Investigation, Data curation.

## Funding statement

This work was funded by the Deanship of Graduate Studies and Scientific Research at Jouf University under grant No. (DGSSR-2025-01-01198).

## Declaration of competing interest

The authors declare that they have no known competing financial interests or personal relationships that could have appeared to influence the work reported in this paper.

## Data Availability

No data was used for the research described in the article.

## Appendix A Supplementary data

Supplementary Table S1. Search strategies used in the review.

Supplementary Table S2. Characteristics and key findings of included studies.
