# Supplementary material for: Early identification and prevention of cardiometabolic risk in Gulf Cooperation Council countries: a scoping review relevant to primary care and family medicine
Source: Prev Med Rep. 2026 Oct 12;71:103662. doi: 10.1016/j.pmedr.2026.103662 (PMC13683712; doi:10.1016/j.pmedr.2026.103662)
Supplement: Supplementary file 1 [file mmc1.docx]

**Supplementary Table S1.** Full electronic search strategies for studies of early identification and prevention of cardiometabolic risk among children, adolescents, adults, and healthcare professionals in Gulf Cooperation Council countries, published January 1, 2020, to February 28, 2026.

| **Database and platform** | **Complete search strategy** | **Records retrieved** |
| --- | --- | --- |
| PubMed/MEDLINE | (("cardiometabolic risk"[Title/Abstract] OR cardiometabolic[Title/Abstract] OR obes*[Title/Abstract] OR obesity[MeSH Terms] OR hypertens*[Title/Abstract] OR hypertension[MeSH Terms] OR dyslipid*[Title/Abstract] OR "metabolic syndrome"[Title/Abstract] OR "metabolic syndrome"[MeSH Terms] OR prediabet*[Title/Abstract] OR "type 2 diabetes"[Title/Abstract] OR "diabetes mellitus, type 2"[MeSH Terms] OR T2DM[Title/Abstract] OR "insulin resistance"[Title/Abstract] OR "insulin resistance"[MeSH Terms]) AND (screen*[Title/Abstract] OR "early identification"[Title/Abstract] OR "early detection"[Title/Abstract] OR "early intervention"[Title/Abstract] OR "risk assessment"[Title/Abstract] OR "risk assessment"[MeSH Terms] OR prevent*[Title/Abstract] OR "risk reduction"[Title/Abstract] OR "health promotion"[Title/Abstract] OR "health promotion"[MeSH Terms] OR "health education"[Title/Abstract] OR "lifestyle intervention"[Title/Abstract] OR "lifestyle modification"[Title/Abstract]) AND ("Gulf Cooperation Council"[Title/Abstract] OR GCC[Title/Abstract] OR "Arab Gulf"[Title/Abstract] OR "Gulf countries"[Title/Abstract] OR "Gulf states"[Title/Abstract] OR "Saudi Arabia"[Title/Abstract] OR "Saudi Arabia"[MeSH Terms] OR "United Arab Emirates"[Title/Abstract] OR "United Arab Emirates"[MeSH Terms] OR UAE[Title/Abstract] OR Kuwait[Title/Abstract] OR Kuwait[MeSH Terms] OR Qatar[Title/Abstract] OR Qatar[MeSH Terms] OR Bahrain[Title/Abstract] OR Bahrain[MeSH Terms] OR Oman[Title/Abstract] OR Oman[MeSH Terms])) AND ("2020/01/01"[Date - Publication] : "2026/02/28"[Date - Publication]) AND English[Language] | 1,451 |
| Scopus | TITLE-ABS-KEY ("cardiometabolic risk" OR cardiometabolic OR obes* OR hypertens* OR dyslipid* OR "metabolic syndrome" OR prediabet* OR "type 2 diabetes" OR T2DM OR "insulin resistance") AND TITLE-ABS-KEY (screen* OR "early identification" OR "early detection" OR "early intervention" OR "risk assessment" OR prevent* OR "risk reduction" OR "health promotion" OR "health education" OR "lifestyle intervention" OR "lifestyle modification") AND TITLE-ABS-KEY ("Gulf Cooperation Council" OR GCC OR "Arab Gulf" OR "Gulf countries" OR "Gulf states" OR "Saudi Arabia" OR "United Arab Emirates" OR UAE OR Kuwait OR Qatar OR Bahrain OR Oman) AND PUBYEAR > 2019 AND PUBYEAR < 2027 AND (LIMIT-TO (LANGUAGE, "English")) | 1,624 |
| Web of Science | TS=("cardiometabolic risk" OR cardiometabolic OR obes* OR hypertens* OR dyslipid* OR "metabolic syndrome" OR prediabet* OR "type 2 diabetes" OR T2DM OR "insulin resistance") AND TS=(screen* OR "early identification" OR "early detection" OR "early intervention" OR "risk assessment" OR prevent* OR "risk reduction" OR "health promotion" OR "health education" OR "lifestyle intervention" OR "lifestyle modification") AND TS=("Gulf Cooperation Council" OR GCC OR "Arab Gulf" OR "Gulf countries" OR "Gulf states" OR "Saudi Arabia" OR "United Arab Emirates" OR UAE OR Kuwait OR Qatar OR Bahrain OR Oman) AND PY=(2020-2026) AND LA=(English) | 989 |

Abbreviations: GCC, Gulf Cooperation Council; LA, language; MeSH, Medical Subject Headings; PY, publication year; T2DM, type 2 diabetes mellitus; TS, topic; UAE, United Arab Emirates.
