# Supplementary material for: Early identification and prevention of cardiometabolic risk in Gulf Cooperation Council countries: a scoping review relevant to primary care and family medicine
Source: Prev Med Rep. 2026 Oct 12;71:103662. doi: 10.1016/j.pmedr.2026.103662 (PMC13683712; doi:10.1016/j.pmedr.2026.103662)
Supplement: Supplementary file 2 [file mmc2.docx]

**Supplementary Table S2.** Study-level characteristics and key findings on early identification and prevention of cardiometabolic risk among children, adolescents, adults, and healthcare professionals in Gulf Cooperation Council countries, with studies published January 2020 to February 2026.

| Author (year) | Country | Study design/setting/population | Assessment | Key findings |
| --- | --- | --- | --- | --- |
| Bahijri et al. (2020) (S1) | Saudi Arabia | Cross-sectional risk-score development study; randomly recruited adult Saudis without diabetes from two-stage cluster sampling; n=1,403 (791 men, 612 women). | Demographics, lifestyle/diet, family history, blood pressure, anthropometry, glycated hemoglobin, fasting glucose and one-hour glucose during an oral glucose tolerance test; logistic regression for non-invasive dysglycemia score. | Dysglycemia prevalence 23.0% (69 diabetes, 259 prediabetes). Risk increased with age to 71% at ≥65 years. Final score area under the receiver operating characteristic curve (AUC) 0.76; sensitivity/specificity ~0.70. |
| Syed et al (2020) (S2) | Qatar | Cross-sectional electronic medical records study in publicly funded primary health centres, Qatar; adults ≥18 years visiting in 2017; 127,941 had complete metabolic syndrome component data. | Age, sex, nationality and five metabolic syndrome components using adapted National Cholesterol Education Program Adult Treatment Panel III criteria; logistic regression. | Overall metabolic syndrome prevalence was 48.8% (62,492/127,941); higher in men (56.7%) than women (42.5%). Prevalence peaked at 50.3% in ages 40–49 years. |
| Alkandari et al (2020) (S3) | Kuwait | National cross-sectional Kuwait Diabetes Epidemiology Program; adults aged ≥20 years; n=4,937. | Demographics, behaviors, medical history, anthropometry and biochemistry; diabetes/prediabetes defined by fasting plasma glucose, glycated hemoglobin and medication history. | Adjusted diabetes prevalence was 19.10% and prediabetes 13.50%. Diabetes prevalence rose from 5.40% at 20–29 years to 64.80% at ≥60 years. |
| Althumiri et al. (2021) (S4) | Saudi Arabia | Nationwide cross-sectional telephone survey across 13 Saudi regions; June 2020; completed interviews n=4,709. | Self-reported weight/height, obesity prevalence by region and sex, and associations with chronic health conditions. | Weighted obesity prevalence was 24.70%. Obesity was associated with type 2 diabetes mellitus (odds ratio [OR] 1.52), hypercholesterolemia (OR 1.69), hypertension (OR 1.61), and sleep apnea (OR 1.82). |
| Mohamad et al (2021) (S5) | United Arab Emirates | Cross-sectional study at United Arab Emirates University, Al Ain; female Emirati young adults aged 17–25 years; n=555. | Anthropometry, blood pressure, fasting plasma glucose and glycated hemoglobin for dysglycemia, plus cardiometabolic biomarkers. | Glycated hemoglobin-based prevalence was 24% for prediabetes and 8.60% for diabetes; fasting plasma glucose-based prediabetes was 9.2%. |
| Mezhal et al. (2021) (S6) | United Arab Emirates | Cross-sectional baseline analysis of United Arab Emirates Healthy Future Study; Emirati adults aged 18–40 years; n=5,126. | Demographics, anthropometry, blood pressure, blood tests, and clustering/accumulation of cardiometabolic risk factors. | About 40% had >2 cardiometabolic risk factors. Obesity was associated with central obesity (adjusted odds ratio [AOR] 4.70), abnormal glycemia (AOR 2.98), hypertension (AOR 3.03) and dyslipidemia (AOR 2.71). |
| Oguoma et al. (2021) (S7) | Kuwait | Population-based cross-sectional survey (Kuwait Diabetes Epidemiology Program, 2011-2014); adults aged 18-82 years with valid records (n=4901). | Body mass index, waist circumference, and sociodemographic/lifestyle correlates. | Overweight 40.60%, obesity 42.10%, and central obesity 73.70%; women had higher odds of obesity and central obesity. |
| Mohammad et al. (2021) (S8) | Kuwait | Cross-sectional study of Kuwaiti adults; 618 men and 620 women (n=1,238), mean age 43.0 years. | Prediabetes and undiagnosed type 2 diabetes mellitus by fasting plasma glucose, two-hour oral glucose tolerance test and glycated hemoglobin; associations with sex, age and body mass index. | Prediabetes prevalence was 47.90% by fasting plasma glucose, 36.80% by two-hour oral glucose tolerance test and 31% by glycated hemoglobin. Undiagnosed type 2 diabetes mellitus was 6.90%, 11% and 4.90%, respectively. |
| Al-Mawali et al. (2021) (S9) | Oman | Large community-based national stepwise approach to noncommunicable disease risk-factor surveillance survey in Oman; adults; n not stated here. | Diabetes, prediabetes and associated sociodemographic, behavioral and metabolic risk factors. | Diabetes prevalence was 15.70% and prediabetes 11.80%. Risk was associated with age, education, raised blood pressure, family history, abnormal waist-to-hip ratio and hypertriglyceridemia. |
| Ahmed et al. (2022) (S10) | Saudi Arabia | Cross-sectional analysis of mixed hypercholesterolemic population in Asir, Saudi Arabia. | Metabolic syndrome/cardiometabolic markers including glucose, glycated hemoglobin, lipid profile, and related biochemical measures by sex, age, and obesity. | Participants showed high metabolic syndrome and cardiometabolic risk, especially with obesity and unhealthy lifestyle patterns. |
| Mahmoud et al. (2022) (S11) | United Arab Emirates | Cross-sectional population-based study in United Arab Emirates; n=3,212 (74.1% men), mean age 39.0±11.3 years. | Metabolic syndrome prevalence and associated sociodemographic risk factors. | Overall metabolic syndrome prevalence was 37.40%; 33.60% in Emiratis and 40.70% in Asian non-Arabs. Higher age, body mass index and marital status were associated with metabolic syndrome. |
| Mezhal et al. (2022) (S12) | United Arab Emirates | Cross-sectional United Arab Emirates Healthy Future Study analysis; participants aged <40 years; n=5,161 (3,196 men, 1,965 women). | Metabolic syndrome prevalence in fasting and non-fasting participants using fasting blood glucose and/or glycated hemoglobin; associations with selected factors. | Age-adjusted metabolic syndrome prevalence was 22.70% in males and 12.50% in females. Age, body mass index and family history were strongly associated with metabolic syndrome. |
| Alkazemi et al. (2022) (S13) | Kuwait | Cross-sectional study in Kuwait; apparently healthy adults without chronic diseases; n=231 (193 women, 38 men). | Serum magnesium, metabolic syndrome and its components using International Diabetes Federation and Adult Treatment Panel III criteria. | Hypomagnesemia was associated with metabolic syndrome and several metabolic syndrome components in apparently healthy adults. |
| Eldakhakhny et al. (2023) (S14) | Saudi Arabia | Cross-sectional study among non-diabetic Saudi adults in Jeddah. | Prevalence of metabolic syndrome and associated demographic/lifestyle factors. | Metabolic syndrome prevalence increased markedly with age, from 5.50% at 20–29 years to 54.30% at 60–69 years. |
| Alawainati et al. (2023) (S15) | Bahrain | Cross-sectional study of adults with type 2 diabetes mellitus in primary care centers in Bahrain. | Body mass index-based obesity status and clinical characteristics among adults with type 2 diabetes mellitus. | 82.40% were overweight/obese; 47.50% obese and 35% overweight; obesity was higher in females and Bahrainis. |
| Althumiri et al (2024) (S16) | Saudi Arabia | Secondary analysis of Sharik Health Indicators Surveillance System cross-sectional telephone surveillance in Saudi Arabia, 2020–2023; Saudi adults; n=92,137. | Obesity prevalence trends and related health, behavioral and psychological factors. | Obesity prevalence fluctuated slightly from 22.20% in 2020 to 21.40% in 2023. |
| Alkahtani et al. (2024) (S17) | Saudi Arabia | Cross-sectional study of middle-aged/older Saudis; men (n=287) and women (n=268). | Anthropometry, body composition, handgrip strength, relative handgrip strength, blood biochemistry, and metabolic syndrome. | Lower handgrip strength quartiles were associated with higher metabolic syndrome risk; adiposity modified the handgrip strength-metabolic syndrome relationship, especially in men. |
| Alsamghan et al. (2025) (S18) | Saudi Arabia | Retrospective cross-sectional hospital-based study in Saudi Arabia. | Body mass index categories and clinical correlates/comorbidities of overweight and obesity. | Overweight/obesity was common; hypertension rates were higher in the obese group, and body mass index correlated with clinical parameters. |
| Abdelgadir et al. (2025) (S19) | United Arab Emirates | Cross-sectional, multicentre, population-based study of adults >18 years attending Dubai Academic Health Corporation (2018-2023). | Prevalence of overweight/obesity using World Health Organization and Asia-Pacific body mass index cut-points; sociodemographic variation. | 63.40% had overweight/obesity; burden was higher in United Arab Emirates nationals, and standard World Health Organization cut-points may underestimate obesity in Southeast Asian groups. |
| Arar et al. (2025) (S20) | Qatar | Cross-sectional Qatar Biobank analysis. | Association between obesity and cholesterol abnormalities. | Obesity was associated with adverse cholesterol profiles in Qatar Biobank participants. |
| Hamran et al. (2025) (S21) | Qatar | Nationally representative cross-sectional study in Qatar. | Sociodemographic/health data; metabolic syndrome by standard criteria; circadian syndrome defined using sleep, depression, and metabolic components. | Metabolic syndrome prevalence was 6.80% and circadian syndrome 2.40%; metabolic syndrome showed stronger associations than circadian syndrome with type 2 diabetes mellitus and cardiovascular diseases. |
| Agwa et al. (2026) (S22) | Saudi Arabia | Cross-sectional study of adults aged >=18 years in Al-Baha, Saudi Arabia (n=1008). | Obesity prevalence and lifestyle factors. | Obesity was highly prevalent, particularly among women and middle-aged adults, supporting targeted lifestyle interventions. |
| Al-Shariqi et al. (2026) (S23) | Oman | Analysis from the national screening program in Muscat Governorate, Oman; adults. | Prediabetes prevalence and associated cardiometabolic risk profiles. | Reported high prediabetes prevalence and associated cardiometabolic risk profiles among Omani adults. |
| Kerkadi et al (2020) (S24) | Qatar | Cross-sectional study of adults from Qatar Biobank. | Anthropometric and dual-energy X-ray absorptiometry-derived adiposity indicators as cardiometabolic risk predictors. | Waist circumference and waist-to-height ratio were clinically useful for identifying adults at cardiometabolic/cardiovascular disease risk. |
| Alkhalaqi et al (2020) (S25) | Qatar | Cross-sectional Qatar Biobank study of adults >20 years; random sample of Qatari nationals and long-term residents; n=1,103. | Compared visceral adiposity index, body adiposity index and body mass index for predicting type 2 diabetes using clinical/anthropometric data. | Visceral adiposity index was a better predictor of type 2 diabetes than body mass index, with stronger association in females. |
| Abbas et al. (2021) (S26) | Qatar | Cross-sectional case-control study using Qatar Biobank cohort; 4,895 controls and 2,373 adults with prediabetes. | Demographic and clinical predictors used to derive the Prediabetes Risk Score in Qatar. | Significant predictors were age, sex, body mass index, waist circumference and blood pressure. Prediabetes Risk Score in Qatar was developed as a simple screening tool for prediabetes. |
| Awad et al. (2022) (S27) | Qatar | Age-structured mathematical modeling study of type 2 diabetes burden in Qatar (2021-2050). | Modeled diabetes burden using obesity, smoking, and physical inactivity as major risk factors. | Type 2 diabetes prevalence was projected to rise from 7% in 2021 to 14% by 2050. |
| Elmagarmid et al. (2024) (S28) | Qatar | Cross-sectional study of Qatari adults using clinical/anthropometric data; n=5,996. | Risk factors for prediabetes in normal-weight adults using machine-learning and clinical variables. | Identified triglycerides as a key risk factor for prediabetes in normal-weight Qatari adults. |
| Sallahi et al. (2024) (S29) | Qatar | Cross-sectional clinical validation study in Qatar primary care; required sample 176 with prediabetes and 528 without; analyzed n=797. | Real-world validation of the Prediabetes Risk Score in Qatar against glycated hemoglobin. | 21.90% had prediabetes and 3.30% had undiagnosed diabetes by glycated hemoglobin; Prediabetes Risk Score in Qatar showed good real-world diagnostic performance. |
| Mezhal et al. (2025) (S30) | United Arab Emirates | Cross-sectional United Arab Emirates Healthy Future Study analysis; final sample n=5,058. | Family history of cardiovascular disease and prevalence of cardiometabolic risk factors in young adults. | 57.60% had a positive family history of cardiovascular disease; family history was associated with higher cardiometabolic risk burden. |
| Mezhal et al. (2023) (S31) | United Arab Emirates | Cross-sectional analysis of baseline United Arab Emirates Healthy Future Study data  Young Emirati adults aged 18–40 years, n = 5,167 | Anthropometry, blood pressure, blood analysis; prevalence/distribution of obesity, dysglycemia, dyslipidemia, hypertension, and central obesity by sociodemographic and behavioral factors | high prevalence of cardiometabolic risk factors; age-adjusted prevalence 26.50% obesity, 11.70% dysglycemia, 62.70% dyslipidemia, 22.40% hypertension, and 22.50% central obesity |
| Al Sabbah H (2020) (S32) | United Arab Emirates | Cross-sectional university study in Dubai, United Arab Emirates; female students; n=251. | Anthropometry, waist circumference, body fat %, hemoglobin, and questionnaire on diet, meals, physical activity and lifestyle. | Nearly one-third were overweight/obese; abdominal obesity 8.50%; Irregular meals were common (71.70%) and highest in obese students. |
| Ibrahim et al. (2020) (S33) | United Arab Emirates | Cross-sectional university study at Ajman University, United Arab Emirates; female undergraduates aged 18–26 years; n=200. | Questionnaire on sociodemographics, diet/lifestyle, medication and family history; weight, height, waist/hip circumference, blood pressure and random glucose. | 20% had body mass index >25 kg/m² and 12% had waist circumference >35 inches. Breakfast skipping was linked to weight gain, higher blood pressure and higher glucose. |
| Al Yazeedi et al. (2020) (S34) | Oman | Cross-sectional study of mother-child dyads from 5 provinces; Omani children aged 6-10 years (n=197). | Body mass index z-scores; dietary recall; questionnaires on nutrition, physical activity, screen time, and sleep. | 17.40% were overweight/obese; longer night sleep was positively associated with obesity, while nutrition, moderate-to-vigorous physical activity, and screen time were not significant. |
| Al-Hazzaa, and Albawardi (2021) (S35) | Saudi Arabia | Cross-sectional study in Riyadh, Saudi Arabia; adolescents aged 14–19 years; n=1,262. | Lifestyle behaviors, dietary habits, body mass index, waist circumference, physical activity and screen time. | Overall overweight/obesity prevalence was 40.50%; higher in males (47.30%) than females (32.80%). |
| Abdelhamed et al. (2022) (S36) | Saudi Arabia | Cross-sectional pediatric obesity study in Saudi Arabia; overweight/obese children and adolescents. | Insulin resistance and adiposity indices as predictors of obesity-related morbidity. | Obesity-related morbidity was present in 61.90%, and insulin resistance in 64.70% of participants. |
| Al-Hazzaa et al. (2022) (S37) | Saudi Arabia | Cross-sectional study from Riyadh and Jeddah, Saudi Arabia; children aged 6–13 years; n=2,169. | Compared body mass index classifications (International Obesity Task Force, World Health Organization and Saudi national references) for overweight/obesity prevalence. | Using the study classification, overweight prevalence was 19.10% and obesity 18.90%. |
| Albaker et al. (2022) (S38) | Saudi Arabia | Cross-sectional study in the Eastern Province, Saudi Arabia; school-age children; n=20,000. | Childhood overweight/obesity prevalence by sex and age group. | Overweight/obesity prevalence was 25.70%; obesity was higher in boys (16.20%) than girls (12.70%). |
| Al-Haifi et al. (2022) (S39) | Kuwait | Cross-sectional study of Kuwaiti adolescents; n=706. | Overweight/obesity prevalence and adolescents’ perception of body weight by parents or friends. | Nearly 50% were overweight/obese; prevalence was higher in males (54.30%) than females (44.60%). |
| AlEnazi et al. (2023) (S40) | Saudi Arabia | Multicenter population-based study in Saudi Arabia; children/adolescents aged 2–19 years; n=351,195. | Prevalence of overweight and obesity by age, sex, nationality and region. | Overall overweight prevalence was 11.20% and obesity 9.40%; obesity was highest at ages 2–6 years (12.30%) and in boys (10.40%). |
| Sharma et al. (2023) (S41) | United Arab Emirates | Cross-sectional study in Al Ain City, United Arab Emirates; youth/university students. | Distribution of cardiometabolic risk factors and their association with excess body weight. | Excess body weight was associated with higher systolic blood pressure, triglycerides and lower high-density lipoprotein cholesterol. |
| Al-Saadi et al. (2023) (S42) | Oman | Cross-sectional study in Al Buraimi Governorate, Oman; 14 government schools; children aged 6–17 years; n=714. | Sociodemographics, lifestyle behaviors, anthropometry and central obesity. | Overweight prevalence was 12.30%, obesity 16.30% and central obesity 21.40%. |
| Alqassimi et al. (2024) (S43) | Saudi Arabia | Cross-sectional study among medical students at Jazan University, Saudi Arabia (n=228). | Questionnaire on sociodemographic, diet, physical activity, lifestyle, and comorbidities; anthropometry including body mass index and waist circumference. | Overweight/obesity burden was notable; most factors were not significant, but family income Saudi riyals 15,000-24,999 was linked with lower obesity odds. |
| Arfaoui et al. (2025) (S44) | Saudi Arabia | Cross-sectional study of female adolescents in Jeddah, Saudi Arabia. | Sociodemographic status, dietary intake, physical activity, and obesity. | Reported elevated obesity prevalence among Saudi female adolescents, supporting school- and community-based interventions. |
| Alfaraidi et al. (2026) (S45) | Saudi Arabia | Cross-sectional study among Saudi adolescents. | Impaired fasting glucose and associated metabolic markers of prediabetes. | Reported impaired fasting glucose prevalence and linked metabolic markers among Saudi adolescents. |
| Althwab et al. (2024) (S46) | Saudi Arabia | Cross-sectional adult study from the Qassim region, Saudi Arabia. | Metabolic syndrome prevalence and atherogenic index of plasma/metabolic predictors of cardiovascular risk. | Atherogenic index of plasma showed potential value for identifying adults at higher cardiometabolic and metabolic syndrome risk. |
| Aldayel et al. (2021) (S47) | Saudi Arabia | Cross-sectional validation study in 5 primary healthcare facilities in Riyadh, Saudi Arabia; adults ≥18 years; n=180. | Arabic version of the American Diabetes Association Prediabetes Risk Test and glycated hemoglobin; sensitivity, specificity, positive predictive value (PPV), negative predictive value (NPV) and AUC. | Sensitivity was 78.90% and specificity 82%. The tool was considered useful for opportunistic prediabetes screening in primary healthcare. |
| Awad et al. (2021) (S48) | Qatar | Mathematical modeling study for Qatari population; representative simulated sample of 5,000 Qataris. | Age, sex, obesity, smoking and physical inactivity were used to derive a diabetes risk score. | Developed a Qatar-specific diabetes risk score to identify individuals at high risk of diabetes. |
| Sadek et al. (2022) (S49) | Qatar | Cross-sectional model development and validation study using Qatar Biobank; derivation sample n=2,000 and validation sample n=1,000. | Developed diabetes and impaired glucose metabolism risk scores using demographic/clinical predictors. | Produced Qatar-specific diabetes and impaired glucose metabolism risk scores to identify high-risk individuals for primary prevention. |
| Al-Lawati et al. (2022) (S50) | Oman | Risk-score development and validation study using Oman World Health Survey 2008 data; n=2,720. | Non-invasive diabetes screening tool using sex, age and other questionnaire-based risk factors. | Developed and validated an updated Omani diabetes risk score for undiagnosed type 2 diabetes mellitus screening. |
| Alshaikh et al. (2024) (S51) | Saudi Arabia | Cross-sectional survey in Aseer region, Saudi Arabia, August 2023; adults. | Arabic version of the Australian Type 2 Diabetes Risk Assessment Tool to predict 5-year risk of diabetes and prediabetes. | 46.20% were predicted to develop impaired glucose tolerance and 21.90% to develop type 2 diabetes mellitus within 5 years. |
| Alshaikhi et al. (2024) (S52) | Saudi Arabia | Community-based screening study in Alqunfudah, Saudi Arabia; adults ≥18 years; n=332. | Anthropometry and community screening for diabetes and prediabetes. | 36.10% were diagnosed with diabetes and 28.30% with prediabetes. |
| Khondaker et al. (2020) (S53) | Qatar | Case-control study using Qatar Biobank data; Qatari adults; n=500 (250 obese, 250 healthy controls). | Clinical biomarkers, spirometry, VICORDER vascular measures, and dual-energy X-ray absorptiometry body-composition data; machine-learning feature selection. | Model accuracy exceeded 90%. Physio-clinical markers were most influential; insulin, C-peptide, uric acid, glycated hemoglobin/glucose and lipid markers distinguished obesity. |
| Alqaderi et al. (2022) (S54) | Kuwait | Longitudinal adolescent cohort analysis from Kuwait Healthy Life Study; adolescents; n=353. | Salivary biomarkers, obesity, intermediate hyperglycemia and longitudinal body mass index change. | Salivary C-reactive protein and insulin were associated with hyperglycemia and obesity, supporting noninvasive risk assessment. |
| Alqaderi et al. (2020) (S55) | Kuwait | Longitudinal adolescent cohort analysis from Kuwait Healthy Life Study; adolescents; n=353. | Salivary biomarkers, obesity, intermediate hyperglycemia and longitudinal body mass index for age percentile change. | Salivary C-reactive protein and insulin were associated with hyperglycemia and obesity, supporting salivary biomarkers as noninvasive risk indicators. |
| Oguoma et al. (2022) (S56) | Kuwait | National cross-sectional analysis from the Kuwait Diabetes Epidemiology Program. | Anthropometric cut-points, especially waist circumference, for screening diabetes and metabolic syndrome in Arab and South Asian groups. | Ethnic-specific anthropometric cut-points were proposed, supporting population-tailored screening for diabetes and metabolic syndrome. |
| Alqabandi et al (2020) (S57) | Kuwait | Text-message-based survey study in Kuwait; 5 campaigns sent to ~180,000 adults (about 6% of adult population). | Six-item type 2 diabetes mellitus risk screen plus Centers for Disease Control and Prevention prediabetes screening; high-risk respondents were invited to a prevention program. | 153 direct responders completed screening; 367 more screened via forwarded messages. Of 520 screened, 121 (23.30%) qualified and agreed to join prevention. |
| Alruways et al. (2020) (S58) | Saudi Arabia | Cross-sectional online survey in Dawadmi, Riyadh province, Saudi Arabia; January–March 2020; adults; n=311. | Sociodemographics, family history, awareness of coronary artery disease risk factors/symptoms/complications, and prevalence of major coronary artery disease risk factors. | 82% had not heard of coronary artery disease; 26.40% were unaware of risk factors; 58.60% were overweight/obese; 79.10% were physically inactive; 24.10% smoked. |
| AlSaleh et al (2021) (S59) | Saudi Arabia | Community-based cross-sectional study in Al-Ahsa region, Saudi Arabia; adult residents; n=812. | Questionnaire on prediabetes knowledge, risk factors and prevention awareness. | 87.10% had high overall knowledge. Age and occupation were significantly associated with knowledge level. |
| Alkaabi et al (2021) (S60) | United Arab Emirates | Pilot interventional study at Diabetes Center, Tawam Hospital, Al Ain, United Arab Emirates; screened 122, enrolled 44 overweight/obese adults with family history of type 2 diabetes mellitus; 32 completed follow-up. | Questionnaire, anthropometry and laboratory tests at baseline, 3 and 6 months after diabetes-prevention education. | At 3 months, 64.50% achieved ≥2 cm waist reduction and 62.50% lost weight; retention was 72.70%. |
| Alqaoud et al. (2021) ^(S61)^ | Kuwait | Repeated cross-sectional analysis of Kuwait national surveys, 2016–2019; preschool children aged 2–5 years; n=5,304. | Trends in overweight/obesity and associations with breakfast skipping, sugary/sweetened beverages and screen time. | Examined 4-year trends in preschool overweight/obesity and identified breakfast skipping, sugar-sweetened beverage intake and screen time as key correlates. |
| Al-Taiar et al. (2021) (S62) | Kuwait | Repeated cross-sectional study using Kuwait Nutrition Surveillance System, 2007–2019; schoolchildren. | Age-specific trends in overweight/obesity using World Health Organization, Centers for Disease Control and Prevention and International Obesity Task Force definitions. | Overweight/obesity increased over 13 years; obesity in girls appeared to plateau during 2014–2019. |
| Almaski and Almughamisi (2025) (S63) | Saudi Arabia | Cross-sectional community study in Al-Madinah, Saudi Arabia; adults surveyed in 2022. | Knowledge of prediabetes and associated risk factors. | Reported low prediabetes knowledge and highlighted the need for community awareness interventions. |
| Alhareky et al. (2024) (S64) | Kuwait | Longitudinal cohort from the Kuwait Healthy Life Study; schoolchildren aged 10-12 years (n=6305). | Beverage intake (soda, juice, milk) and incident obesity over 2 years. | 6% developed obesity; high soda intake was significantly associated with incident obesity; high milk intake also showed a significant association. |
| Barakat et al. (2024) (S65) | Bahrain | Cross-sectional observational study among adults attending 8 public healthcare centres (n=356). | Questionnaire-based obesity perceptions plus measured weight and height. | Obesity prevalence was 41.40%; only 49% of obese participants perceived themselves as obese, and 45.80% considered obesity a disease. |
| Al-Wawi et al (2025) (S66) | United Arab Emirates | Cross-sectional study among United Arab Emirates adults, using convenience sampling. | Prediabetes knowledge, attitudes, practices, and risk levels. | Knowledge and health practices were generally insufficient despite positive attitudes toward prediabetes prevention. |
| AlTaib et al. (2026) (S67) | Saudi Arabia | Retrospective cohort at lifestyle medicine clinics in primary care, Prince Sultan Military Medical City, Riyadh (2023-2024); adults with obesity (n=664; 280 matched pairs). | Anthropometric and metabolic profile; comparison of liraglutide plus lifestyle modification vs lifestyle modification alone. | Both groups improved body mass index, blood pressure, and glycated hemoglobin; added liraglutide benefit was modest and mainly limited to waist circumference. |
| Jareebi et al. (2025) (S68) | Saudi Arabia | Analytical cross-sectional study in Jazan region, Saudi Arabia, May–June 2024; adults ≥18 years; n=3,411. | Modifiable and non-modifiable determinants of diabetes mellitus. | Findings supported integrated, culturally tailored prevention and early screening for higher-risk adults. |
| Cheema et al. (2022) (S69) | Qatar | Cross-sectional study among adolescents in Qatar, 2018–2020; n=459. | Overweight, obesity, central obesity and random blood sugar; associations with early-life/lifestyle factors. | 23.40% were overweight, 19.90% obese and 37.60% centrally obese; 5% had random glucose suggestive of prediabetes. |
| Mohieldein et al. (2023) (S70) | Saudi Arabia | Cross-sectional study of school-aged children in Qassim, Saudi Arabia. | Dietary patterns, physical activity and prevalence of overweight/obesity. | Assessed healthy behaviors and overweight/obesity prevalence among school-aged children. |
| Haroun and Ehsanallah et al. (2024) (S71) | United Arab Emirates | Cross-sectional study of 131 female university students aged 17-27 years. | Self-reported and measured weight/height; body mass index agreement analyses. | 87% had self-reported body mass index within the correct category; agreement was strong (kappa=0.87), with slight body mass index underestimation. |
| Kutbi and Mumena (2025) (S72) | Saudi Arabia | Multi-school cross-sectional study of Saudi adolescents. | Prevalence of overweight/obesity and associations with parental weight status and lifestyle/socioeconomic factors. | Parental overweight/obesity was common; adolescent overweight/obesity was associated with parental weight status. |
| Salim et al. (2025) (S73) | Oman | Cross-sectional study among Omani adolescents with obesity. | Metabolic syndrome prevalence and fitness-related predictive risk factors. | Reported prevalence and predictive fitness components of metabolic syndrome among adolescents with obesity. |
| Mondal et al (2025) (S74) | Oman | Cross-sectional study among college students in Oman. | Obesity prevalence and associated implications for health and academic performance. | Highlighted obesity burden among Omani college students and its health/academic implications. |
| Tourkmani et al. (2024) (S75) | Saudi Arabia | Cross-sectional study in Saudi Arabia | Characteristics and risk factors associated with prediabetes development. | Identified several factors associated with development of prediabetes in the Saudi population. |
| Al-Harrasi et al. (2022) (S76) | Oman | Structural equation modeling study using national survey data from Oman; adult population. | Direct and indirect sociodemographic, behavioral and metabolic risk factors of diabetes. | Eight direct paths to diabetes status were identified, including age, sex, education, physical activity, body mass index, waist-to-hip ratio, systolic blood pressure and family history. |
| Alyahya and Rabbani (2025) (S77) | Saudi Arabia | Cross-sectional online survey of primary care physicians in Qassim, Saudi Arabia. | Knowledge, attitudes and practices regarding prediabetes screening and management. | Mean scores were 3.41/5 for knowledge, 12.84/16 for attitude and 3.38/5 for practice; family physicians scored higher than general practitioners. |
| Almubark et al. (2022) (S78) | Saudi Arabia | Nationwide descriptive cross-sectional study in Saudi Arabia; adults ≥18 years; n=14,007. | Sociodemographic and behavioral characteristics among adults with diabetes. | Diabetes prevalence was 11.70% (1,633/14,007). Only 7% of diabetic participants reported adequate fruit/vegetable intake. |
| Aljehani et al. (2022) (S79) | Saudi Arabia | Cross-sectional online survey of primary care physicians in Saudi Arabia. | Prediabetes screening and treatment approaches, knowledge of risk factors and management opinions. | Reported significant knowledge gaps and variation in screening and treatment approaches for prediabetes. |
| Nasaif et al. (2024) (S80) | Bahrain | Cross-sectional secondary-data study of nurses in 3 Bahrain hospitals (n=550). | Body mass index-based prevalence of overweight/obesity and associated demographic factors. | Overweight/obesity was common among nurses, highlighting the need for workplace health promotion. |

**Supplementary References:**

S1.	Bahijri S, Al-Raddadi R, Ajabnoor G, Jambi H, Al Ahmadi J, Borai A, et al. Dysglycemia risk score in Saudi Arabia: A tool to identify people at high future risk of developing type 2 diabetes. Journal of Diabetes Investigation. 2020;11(4):844-55.

S2.	Syed MA, Al Nuaimi AS, Latif Zainel AJA, A/Qotba HA. Prevalence of metabolic syndrome in primary health settings in Qatar: a cross sectional study. BMC Public Health. 2020;20(1):611.

S3.	Alkandari A, Alarouj M, Elkum N, Sharma P, Devarajan S, Abu-Farha M, et al. Adult Diabetes and Prediabetes Prevalence in Kuwait: Data from the Cross-Sectional Kuwait Diabetes Epidemiology Program. Journal of Clinical Medicine [Internet]. 2020; 9(11):[3420 p.].

S4.	Althumiri NA, Basyouni MH, AlMousa N, AlJuwaysim MF, Almubark RA, BinDhim NF, et al. Obesity in Saudi Arabia in 2020: Prevalence, Distribution, and Its Current Association with Various Health Conditions. Healthcare [Internet]. 2021; 9(3):[311 p.].

S5.	Mohamad MN, Ismail LC, Stojanovska L, Apostolopoulos V, Feehan J, Jarrar AH, et al. The prevalence of diabetes amongst young Emirati female adults in the United Arab Emirates: A cross-sectional study. PLOS ONE. 2021;16(6):e0252884.

S6.	Mezhal F, Oulhaj A, Abdulle A, AlJunaibi A, Alnaeemi A, Ahmad A, et al. The interrelationship and accumulation of cardiometabolic risk factors amongst young adults in the United Arab Emirates: The UAE Healthy Future Study. Diabetology & Metabolic Syndrome. 2021;13(1):140.

S7.	Oguoma VM, Coffee NT, Alsharrah S, Abu-Farha M, Al-Refaei FH, Al-Mulla F, et al. Prevalence of overweight and obesity, and associations with socio-demographic factors in Kuwait. BMC Public Health. 2021;21(1):667.

S8.	Mohammad A, Ziyab AH, Mohammad T. Prevalence of Prediabetes and Undiagnosed Diabetes Among Kuwaiti Adults: A Cross-Sectional Study. Diabetes, Metabolic Syndrome and Obesity: Targets and Therapy. 2021;14(null):2167-76.

S9.	Al-Mawali A, Al-Harrasi A, Jayapal SK, Morsi M, Pinto AD, Al-Shekaili W, et al. Prevalence and risk factors of diabetes in a large community-based study in the Sultanate of Oman: STEPS survey 2017. BMC Endocr Disord. 2021;21(1):42.

S10.	Ahmed AE, Alsamghan A, Momenah MA, Alqhtani HA, Aldawood NA, Alshehri MA, et al. Metabolic Syndrome and Cardiometabolic Risk Factors in the Mixed Hypercholesterolemic Populations with Respect to Gender, Age, and Obesity in Asir, Saudi Arabia. Int J Environ Res Public Health. 2022;19(22).

S11.	Mahmoud I, Sulaiman N. Prevalence of Metabolic Syndrome and Associated Risk Factors in the United Arab Emirates: A Cross-Sectional Population-Based Study. Frontiers in Public Health. 2022;Volume 9 - 2021.

S12.	Mezhal F, Ahmad A, Abdulle A, Leinberger-Jabari A, Oulhaj A, AlJunaibi A, et al. Metabolic Syndrome in Fasting and Non-Fasting Participants: The UAE Healthy Future Study. International Journal of Environmental Research and Public Health [Internet]. 2022; 19(21):[13757 p.].

S13.	Alkazemi D, Alsouri N, Zafar T, Kubow S. Hypomagnesemia and the Metabolic Syndrome among Apparently Healthy Kuwaiti Adults: A Cross-Sectional Study. Nutrients [Internet]. 2022; 14(24):[5257 p.].

S14.	Eldakhakhny B, Enani S, Jambi H, Ajabnoor G, Al-Ahmadi J, Al-Raddadi R, et al. Prevalence and Factors Associated with Metabolic Syndrome among Non-Diabetic Saudi Adults: A Cross-Sectional Study. Biomedicines [Internet]. 2023; 11(12):[3242 p.].

S15.	Alawainati MA, Ayoob ZA, AlQari AA, Makhlooq F, Naser HS, Bukamal F. Prevalence and characteristics of obesity in patients with type-2 diabetes mellitus in primary care centers in Bahrain: A cross-sectional study. J Family Community Med. 2023;30(2):109-15.

S16.	Althumiri NA, Bindhim NF, Al-Rayes SA, Alumran A. Mapping Obesity Trends in Saudi Arabia: A Four-Year Description Study. Healthcare [Internet]. 2024; 12(20):[2092 p.].

S17.	Alkahtani SA, Alshammari GM, Alzuwaydi A, Alfuhaid A, Al-Masri AA, Qaisar R, et al. Association between handgrip strength and metabolic syndrome in relation to gender and adiposity among middle aged and older Saudi populations. Aging Male. 2024;27(1):2325146.

S18.	Alsamghan A, Mahmood SE, Ahmad A, Alsaleem MA. Prevalence and clinical correlates of overweight and obesity among adults in Abha, Saudi Arabia: A retrospective cross-sectional study. Medicine (Baltimore). 2025;104(48):e45804.

S19.	Abdelgadir E, Rashid F, Bashier A, Zidan M, McGowan B, Alawadi F. Prevalence of overweight and obesity in adults from the Middle East: A large-scale population-based study. Diabetes, Obesity and Metabolism. 2025;27(7):3676-85.

S20.	Arar N, Ayon N, Hashem R, Alhussaini N, Abdel-Rahman ME. Obesity and cholesterol: A cross-sectional analysis of Qatar Biobank data. Qatar Med J. 2025;2025(4):97.

S21.	Hamran S, Altrmanini O, Rahhal MO, Alsheikh R, Amro I, Babu GR, et al. Associations of Circadian and Metabolic Syndromes with Cardiovascular Diseases and Diabetes in Qatar: A Cross-Sectional Study. Diabetes, Metabolic Syndrome and Obesity: Targets and Therapy. 2025;18(null):3259-69.

S22.	Agwa RH, Alkully TH, Alghamdi TA, Alghamdi RF, Alzahrani AM, Alghamdi WS, et al. Prevalence of obesity and its relationship with lifestyle patterns among adults in Al-Baha, Saudi Arabia: A cross-sectional study. Medicine (Baltimore). 2026;105(3):e47038.

S23.	Al-Shariqi FT, Al-Mazrooei S, Al-Harrasi A, Ismail M, Al-Kathiri F, Al-Ismaili M, et al. High Prevalence of Prediabetes and Cardiometabolic Risk Profiles Among Omani Adults in the Muscat Governorate: Analysis from the National Screening Program. Journal of the Oman Medical Association [Internet]. 2026; 3(1):[4 p.].

S24.	Kerkadi A, Suleman D, Abu Salah L, Lotfy C, Attieh G, Bawadi H, et al. Adiposity Indicators as Cardio-Metabolic Risk Predictors in Adults from Country with High Burden of Obesity. Diabetes, Metabolic Syndrome and Obesity: Targets and Therapy. 2020;13(null):175-83.

S25.	Alkhalaqi A, Al-Naimi F, Qassmi R, Shi Z, Ganji V, Salih R, et al. Visceral adiposity index is a better predictor of type 2 diabetes than body mass index in Qatari population. Medicine. 2020;99(35).

S26.	Abbas M, Mall R, Errafii K, Lattab A, Ullah E, Bensmail H, et al. Simple risk score to screen for prediabetes: A cross-sectional study from the Qatar Biobank cohort. Journal of Diabetes Investigation. 2021;12(6):988-97.

S27.	Awad SF, A Toumi A, A Al-Mutawaa K, A Alyafei S, A Ijaz M, A H Khalifa S, et al. Type 2 diabetes epidemic and key risk factors in Qatar: a mathematical modeling analysis. BMJ Open Diabetes Research & Care. 2022;10(2):e002704.

S28.	Elmagarmid KA, Fadlalla M, Jose J, Arredouani A, Bensmail H. Investigation of the risk factors associated with prediabetes in normal-weight Qatari adults: a cross-sectional study. Scientific Reports. 2024;14(1):23116.

S29.	Sallahi N, Zainel AA, Bensmail HN, Syed MA, Arredouani A. Real-world clinical validation of the Qatar pre-diabetes risk score: a cross-sectional study. BMJ Public Health. 2024;2(1):e000957.

S30.	Mezhal F, Ahmad A, Abdulle A, Leinberger-Jabari A, AlJunaibi A, Alnaeemi A, et al. Association of family history of cardiovascular disease with the prevalence of cardiometabolic risk factors in young adults in the United Arab Emirates: The UAE healthy future study. PLOS ONE. 2025;20(3):e0319648.

S31.	Mezhal F, Oulhaj A, Abdulle A, AlJunaibi A, Alnaeemi A, Ahmad A, et al. High prevalence of cardiometabolic risk factors amongst young adults in the United Arab Emirates: the UAE Healthy Future Study. BMC Cardiovasc Disord. 2023;23(1):137.

S32.	Al Sabbah H. Prevalence of overweight/obesity, anaemia and their associations among female university students in Dubai, United Arab Emirates: a cross-sectional study. Journal of Nutritional Science. 2020;9:e26.

S33.	Ibrahim NA, Saleh NM, Koprulu F, Abdulrahim AH. Metabolic Syndrome associated Risk factors: Findings among female undergraduate university students. Research Journal of Pharmacy and Technology. 2020;13(12):6093-7.

S34.	Al Yazeedi B, Berry DC, Crandell J, Waly M. Lifestyle-Related Determinants of Obesity Among Omani Children. Sultan Qaboos Univ Med J. 2020;20(3):e279-e86.

S35.	Al-Hazzaa HM, Albawardi NM. Obesity, Lifestyle Behaviors, and Dietary Habits of Saudi Adolescents Living in Riyadh (ATLS-2 Project): Revisited after a Ten-Year Period. Life [Internet]. 2021; 11(10):[1078 p.].

S36.	Abdelhamed MH, Salah S, KK AL, Jan MM, Alahdal DK, Alfaifi SA, et al. Indices of insulin resistance and adiposity can detect obesity-related morbidity in pediatrics. Saudi Med J. 2022;43(2):161-8.

S37.	Al-Hazzaa HM, Alrasheedi AA, Alsulaimani RA, Jabri L, Alhowikan AM, Alhussain MH, et al. Prevalence of overweight and obesity among saudi children: A comparison of two widely used international standards and the national growth references. Frontiers in Endocrinology. 2022;Volume 13 - 2022.

S38.	Albaker W, Saklawi R, Bah S, Motawei K, Futa B, Al-Hariri M. What is the current status of childhood obesity in Saudi Arabia?: Evidence from 20,000 cases in the Eastern Province: A cross-sectional study. Medicine. 2022;101(27).

S39.	Al-Haifi AR, Al-Awadhi BA, Al-Dashti YA, Aljazzaf BH, Allafi AR, Al-Mannai MA, et al. Prevalence of overweight and obesity among Kuwaiti adolescents and the perception of body weight by parents or friends. PLOS ONE. 2022;17(1):e0262101.

S40.	AlEnazi S, AlAjlan R, AlKhalaf H, Abolfotouh M, Alharbi O, Alfawaz R, et al. Prevalence of Obesity among Children and Adolescents in Saudi Arabia: A Multicenter Population-Based Study. Saudi J Med Med Sci. 2023;11(1):19-25.

S41.	Sharma C, Hassan A, Hamad SA, Yasin J, Alkaabi J, Aburawi EH. Distribution and Association of Cardiometabolic Risk Factors Among Youth From Al Ain City, United Arab Emirates. Cureus. 2023;15(11):e48681.

S42.	Al-Saadi A, Al Yahiaee L, Ahmed E, Al Manee R, Al Saadi L, Mohamed N, et al. Obesity and lifestyle behaviours among in-school children in Oman. East Mediterr Health J. 2023;29(9):716-24.

S43.	Alqassimi S, Elmakki E, Areeshi AS, Aburasain AB, Majrabi AH, Masmali EM, et al. Overweight, Obesity, and Associated Risk Factors among Students at the Faculty of Medicine, Jazan University. Medicina [Internet]. 2024; 60(6):[940 p.].

S44.	Arfaoui L, Alghanmi A, Alamri R, Aljehani N, Alkhaldy A, Assidi M. Sociodemographic Status, Dietary Intake, and Physical Activity in Relation to Body Mass Index Among Female Adolescents in Saudi Arabia. Children [Internet]. 2025; 12(7):[823 p.].

S45.	Alfaraidi H, Babiker A, Al Juraibah F, Tamimi W, Al Dubayee M, Badri M, et al. Prevalence of Impaired Fasting Glucose (IFG) and Associated Prediabetes Metabolic Markers Among Adolescents in Saudi Arabia. Pediatric Diabetes. 2026;2026(1):5568644.

S46.	Althwab SA, Abdulmonem WA, Allemailem KS, Alarifi SA, Hamad EM. Prediction of cardiovascular risk factors and metabolic syndrome in adults from Saudi Arabia using the logarithm of triglyceride/HDL-cholesterol ratio. Int J Health Sci (Qassim). 2024;18(2):50-5.

S47.	Aldayel FA, Belal MA, Alsheikh AM. The Validity of the American Diabetes Association's Diabetes Risk Test in a Saudi Arabian Population. Cureus. 2021;13(9):e18018.

S48.	Awad SF, Dargham SR, Toumi AA, Dumit EM, El-Nahas KG, Al-Hamaq AO, et al. A diabetes risk score for Qatar utilizing a novel mathematical modeling approach to identify individuals at high risk for diabetes. Scientific Reports. 2021;11(1):1811.

S49.	Sadek K, Abdelhafez I, Al-Hashimi I, Al-Shafi W, Tarmizi F, Al-Marri H, et al. Screening for diabetes and impaired glucose metabolism in Qatar: Models’ development and validation. Primary Care Diabetes. 2022;16(1):69-77.

S50.	Al-Lawati NA, Alfonso H, Al-Lawati J. Development and Validation of a Risk Score for Diabetes Screening in Oman. Oman Med J. 2022;37(1):e340.

S51.	Alshaikh AA, Al-Qahtani FS, Taresh HM, Hayaza RA, Alqhtani SS, Summan SI, et al. Prediction of Diabetes and Prediabetes among the Saudi Population Using a Non-Invasive Tool (AUSDRISK). Medicina [Internet]. 2024; 60(5):[775 p.].

S52.	Alshaikhi SA, Alamri AM, Alzilai IY, Alghanimi AA, Alrufaidi AM, Alrufaidi AM, et al. Diabetes and prediabetes prevalence through a community-based screening initiative in Alqunfudah, Saudi Arabia. Future Science OA. 2024;10(1):FSO946.

S53.	Khondaker MT, Khan JY, Refaee MA, Hajj NE, Rahman MS, Alam T. Obesity in Qatar: A Case-Control Study on the Identification of Associated Risk Factors. Diagnostics [Internet]. 2020; 10(11):[883 p.].

S54.	Alqaderi H, Hegazi F, Al-Mulla F, Chiu C-J, Kantarci A, Al-Ozairi E, et al. Salivary Biomarkers as Predictors of Obesity and Intermediate Hyperglycemia in Adolescents. Frontiers in Public Health. 2022;Volume 10 - 2022.

S55.	Alqaderi H, Al-Ozairi E, Bin-Hasan S, Tavares M, Goodson Jo M, Alsumait A, et al. Mediation Effect of C-Reactive Protein in the Relationship Between Abdominal Obesity and Intermediate Hyperglycemia in Kuwaiti Adolescents. Biomarkers in Medicine. 2020;14(15):1427-37.

S56.	Oguoma VM, Coffee NT, Alsharrah S, Abu-Farha M, Al-Refaei FH, Alkandari A, et al. Anthropometric cut-points for discriminating diabetes and the metabolic syndrome among Arabs and Asians: the Kuwait Diabetes Epidemiology Program. British Journal of Nutrition. 2022;127(1):92-102.

S57.	Alqabandi N, Al-Ozairi E, Ahmed A, Ross EL, Jamison RN. Secondary Impact of Social Media via Text Message Screening for Type 2 Diabetes Risk in Kuwait: Survey Study. JMIR Diabetes. 2020;5(4):e20532.

S58.	Alruways AFH, Alotaibi NA, Rashikh MA, Alnufeie AA, Alshammari YJD, Alharthy MR, et al. Awareness and prevalence of coronary artery disease risk factors among Saudi adults in Dawadmi, Riyadh province: A cross-sectional study. Journal of Family Medicine and Primary Care. 2020;9(11).

S59.	AlSaleh E, Alnaser M, Alsalman A, Alramdhan A, Alsalman H, Alhamrani O, et al. A Community-Based Prediabetes Knowledge Assessment among Saudi Adults in Al-Ahsa Region, 2018. Journal of Public Health Research. 2021;10(1):jphr.2021.32.

S60.	Alkaabi JM, Al-Maskari F, Afandi B, Yousef S, Shah SM, Heideman WH, et al. Effects of Diabetes Prevention Education Program for Overweight and Obese Subjects with a Family History of Type 2 Diabetes Mellitus: A Pilot Study from the United Arab Emirates. Oman Med J. 2021;36(3):e268.

S61.	Alqaoud N, Al-Jawaldeh A, Al-Anazi F, Subhakaran M, Doggui R. Trend and Causes of Overweight and Obesity among Pre-School Children in Kuwait. Children [Internet]. 2021; 8(6):[524 p.].

S62.	Al-Taiar A, Alqaoud N, Ziyab AH, Alanezi F, Subhakaran M, Sharaf Alddin R, et al. Time trends of overweight and obesity among schoolchildren in Kuwait over a 13-year period (2007–2019): repeated cross-sectional study. Public Health Nutrition. 2021;24(16):5318-28.

S63.	Almaski A, Almughamisi M. Assessment of prediabetes knowledge among adults in Al-Madinah, Saudi Arabia. Nutrition and Health. 2025;31(1):133-40.

S64.	Alhareky M, Goodson JM, Tavares M, Hartman M-L. Beverage consumption and obesity in Kuwaiti school children. Frontiers in Endocrinology. 2024;Volume 14 - 2023.

S65.	Barakat O, Babili M, AlFaraj N, Almusalam R, Swartz S, Al-Hussain BN, et al. Bodyweight and obesity perceptions among adults in Bahrain. East Mediterr Health J. 2024;30(3):196-203.

S66.	Al-Wawi O, Alameleh H, Alhaj Ahmad M, Al Shamsi F, Suwan L, Bin Kowayer J, et al. Prediabetes Knowledge, Attitudes, Practices, and Risk Levels in the United Arab Emirates: A Cross-Sectional Study. Cureus. 2025;17(4):e82099.

S67.	AlTaib HN, AlAqeel R, Arafat AA, Kunnathodi F, AlShaikh A, AlOtaibi HF. Clinical and metabolic profile of adults with obesity attending lifestyle medicine clinics. PLOS ONE. 2026;21(2):e0342153.

S68.	Jareebi MA, Gosadi IM. Interplay of Modifiable and Non-Modifiable Risk Factors for Diabetes Mellitus in Saudi Adults. Diagnostics [Internet]. 2025; 15(19):[2451 p.].

S69.	Cheema S, Abraham A, El-Nahas KG, Abou-Amona R, Al-Hamaq AO, Maisonneuve P, et al. Assessment of Overweight, Obesity, Central Obesity, and Type 2 Diabetes among Adolescents in Qatar: A Cross-Sectional Study. International Journal of Environmental Research and Public Health [Internet]. 2022; 19(21):[14601 p.].

S70.	Mohieldein A, Elhabiby M, Abu Mustafa A, Shehade M, Alsuhaibani S. A Cross-Sectional Analysis of Health Behavior in School-Aged Children: The Qassim Study. Cureus. 2023;15(11):e48420.

S71.	Haroun D, Ehsanallah A. Validity of self-reported weight and height among female young adults in the United Arab Emirates. PLOS ONE. 2024;19(4):e0302439.

S72.	Kutbi HA, Mumena WA. Overweight and obesity among adolescents in Saudi Arabia: a multi-school cross-sectional study. BMC Pediatrics. 2025;25(1):350.

S73.	Salim AY, Abadi FH, Abdullah NF, Mahfoodha AK, Rahim NA. The Prevalence and Predictive Risk Factors of Metabolic Syndrome Based on Health-Related Fitness Components in Omani Adolescents with Obesity. Iran J Public Health. 2025;54(5):1024-33.

S74.	Mondal S, Basu C, Alkhawaitri M, Almamari I, Albrwaney S, Alhabsi T. Obesity among college students in Oman: implications for health and academic performance. BMC Public Health. 2025;25(1):1111.

S75.	Tourkmani AM, Alharbi TJ, Bin Rsheed AM, Alotaibi AF, AlEisaa M, Youzghadli IM, et al. Characteristics and risk factors associated with developing prediabetes in Saudi Arabia. Annals of Medicine. 2024;56(1):2413922.

S76.	Al-Harrasi A, Pinto AD, Jayapal SK, Morsi M, Al-Mawali A. Structural equation modeling to identify the direct and indirect risk factors of diabetes in adults: Findings from a national survey. The American Journal of the Medical Sciences. 2022;364(3):274-80.

S77.	Alyahya AK, Rabbani U. Assessment of the Knowledge, Attitudes, and Practices of Primary Care Physicians Toward Prediabetes in Qassim, Saudi Arabia. Cureus. 2025;17(7):e87454.

S78.	Almubark RA, Althumairi NA, Alhamdan AA, AlNujaidi HY, Alzayer R, Almubark SA, et al. Socioeconomic and Behavioral Disparities Among Diabetics in Saudi Arabia: A Nation-Wide Descriptive Study. Diabetes, Metabolic Syndrome and Obesity: Targets and Therapy. 2022;15(null):2693-703.

S79.	Aljehani F, Alsulaiman A, Alqarni A, Almutairi F, Samkari M. Survey of Primary Care Physicians' Screening and Treatment Practices for Prediabetes in Saudi Arabia. Cureus. 2022;14(1):e21475.

S80.	Nasaif H, Alaradi M, Hammam R. Prevalence of overweight and obesity among nurses in Bahrain: A cross-sectional study. Nursing Open. 2024;11(1):e2090.
